# Real-world data of docetaxel-induced liver injury among hospitalized patients in Wuhan, China: A retrospective analysis

**DOI:** 10.1097/MD.0000000000042881

**Published:** 2025-06-20

**Authors:** Haiyan Zhan, Yin Deng, Caifei Huang, Jing Xiang Yang, Jian Yang

**Affiliations:** a Department of Pharmacy, Renmin Hospital of Wuhan University, Wuhan, China; b Department of Pharmacy, Wuhan Jinyintan Hospital, Wuhan, China; c Department of Pharmacy, Wuhan Children’s Hospital, Tongji Medical College, Huazhong University of Science & Technology, Wuhan, China.

**Keywords:** docetaxel, incidence, liver injury, risk factor, therapeutic regimen

## Abstract

This study aims to analyze the risk factors of docetaxel-induced liver injury to provide evidence for the clinical prevention and treatment of docetaxel-induced liver injury. A retrospective analysis of patients who received docetaxel chemotherapy regimen from January 2017 to April 2018 in Renmin Hospital of Wuhan University was conducted. Univariate analysis and multivariate logistic regression analysis with the forward stepwise method were used to assess the risk factors associated with liver injury induced by docetaxel. Receiver-operator characteristic curve analysis was performed to calculate the area under the receiver-operator characteristic curve (AUC). In the study, 223 (7.88%) patients were diagnosed as docetaxel-induced liver injury, among which the patients with ovarian cancer had the highest incidence rate (8.33%). By logistic regression analysis, hepatitis B virus carrier, diabetes, docetaxel plus nedaplatin, docetaxel plus capecitabine, docetaxel plus epirubicin, and docetaxel plus cyclophosphamide chemotherapy regimens, were independently associated with drug-induced liver injury during receiving chemotherapy, respectively. Among them, diabetes and docetaxel plus cyclophosphamide were protective factors, but the others were risk factors. Further analysis by the risk score and AUC showed that those factors contributed to an AUC of 0.693 (95% confidence interval = 0.660–0.727), with a predictive sensitivity of 70.9% and specificity of 61.6%. Docetaxel-induced liver injury with a relatively higher incidence should be addressed among ovary cancer patients. The predominant risk factors of docetaxel-induced liver injury included hepatitis B virus carrier and docetaxel combination regimens, and the protective factor was diabetic patients. Among these therapeutic combination regimens, docetaxel plus epirubicin, docetaxel plus nedaplatin, and docetaxel plus capecitabine could significantly increase the occurrence of docetaxel-induced liver injury during hospitalization respectively, while docetaxel plus cyclophosphamide regime might be safer. Therefore, for patients with these risk factors, it might be advisable to actively consider optimum management to reduce the occurrence of docetaxel-induced liver injury during hospitalization, particularly hepatic function test.

## 1. Introduction

Drug-induced liver injury is emerging as one of the most common liver diseases in clinical practice. Individual drug-induced liver injury (DILI) susceptibility depends on patient’s characteristics, host genetic background, disease state, concomitant medications, and immunological and environmental factors.^[1,2]^ Actually, DILI accounts for most cases of acute liver failure with a fatality rate of up to 50%,^[3]^ which highlights the importance of a better understanding of this disease, given that DILI is preventable.

Docetaxel is an antineoplastic agent that inhibits cellular mitosis, playing a central role in treating solid tumors such as breast and lung cancer.^[4]^ Although docetaxel has inspiring therapeutic effects, undesirable side effects are unavoidable, especially for the liver injury that involved in many patients during the process of chemotherapy. More than 10% of Chinese patients with metastatic breast cancer receiving docetaxel monotherapy had suffered from liver injury.^[5]^ There is increasingly awareness for the occurrence of docetaxel-induced liver injury, although the underlying mechanism of occurrence and its risk factors remain unclear. Because of the increased awareness of docetaxel-induced liver injury and the potential risk, resulting in fatal complications for cancer patients, a few groups have sought to determine the prevalence of clinical DILI. It was not eligible for those investigators to gain the actual epidemiology data for the patients. It is of great significance to study the potential risk factors that different chemotherapy regimens play and develop effective therapies for the DILI during the docetaxel treatments.

Recently, very few studies further explored the risk factors of docetaxel-induced liver injury by combining data of socio-demographic characteristics, clinical characteristics and laboratory data. Therefore, we aimed to analyze the incidence and associated risk factors of docetaxel-induced liver injury in Renmin Hospital of Wuhan University, and to provide evidence for clinical prevention and treatment of docetaxel-induced liver injury.

## 2. Materials and methods

### 2.1. Study design and participants

The medical records of 3094 patients receiving docetaxel therapy at Renmin Hospital of Wuhan University from January 2017 and April 2018 were collected. 108 patients had been excluded due to incomplete drug record, the remaining 2986 patients, including 746 males and 2240 females, were enrolled in the study.

They were prescribed docetaxel concurrently during hospitalization. The inclusion criteria were as follows: (1) hospitalization for at least 7 days; (2) normal range of liver function test (LFT) at baseline; (3) conduction of follow-up laboratory data adequate for assessing liver injury; (4) prescription of docetaxel. Excluded from this study were patients who had evidence of liver injury according to the criteria of the Council for International Organization of Medical Science^[6]^ or based on initial LFT results, or known liver dysfunction caused by liver diseases, such as acute viral hepatitis (including hepatitis A virus, hepatitis B virus [HBV], and hepatitis C virus), liver cirrhosis, hepatocellular carcinoma, and so on, at admission. We also excluded cases with incomplete data.

We retrospectively reviewed all serum laboratory data that were collected from inpatient records between January 2017 and April 2018. Our search through individual review of their electronic medical records to determine whether inpatients met the inclusion and exclusion criteria. LFTs, including alanine aminotransferase (ALT), aspartate aminotransferase (AST), alkaline phosphatase (ALP), gamma-glutamyl transferase, and total bilirubin (TBIL), at both admission and follow-up, were reviewed. Patients were divided into 2 groups of DILI and non-DILI.

### 2.2. Liver test abnormalities and liver injury

Liver test abnormalities were defined as the elevation of the following liver enzymes in serum: ALT > 40 U/L, AST > 40 U/L, ALP > 100 U/L, gamma-glutamyl transferase > 45 U/L, and TBIL > 23 µmol/L.

Acute liver injury was defined on biological criteria based on the upper limit of the normal range (ULN) of ALT, ALP, and TBIL, as follows^[6]^: (1) ALT equal or >5 × ULN; (2) ALT equal or >3 × ULN, and total bilirubin >2 × ULN, and no or minimal elevations in ALP; (3) ALP equal or >2 × ULN when the source of increased ALP levels is the liver.

Acute liver injury was classified into 3 types according to the ratio (R = value (ALT/ULN)/(ALP/ULN)) of ALT activity to ALP activity expressed as multiples of ULN^[6]^: (1) hepatocellular injury: *R* ≥ 5, or ALT > 2 × ULN and ALP in normal range; (2) cholestatic injury: *R* ≤ 2, or ALP > 2 × ULN and ALT in normal range; (3) hepatocellular–cholestatic mixed injury: ALT > 2 × ULN, ALP > 1 × ULN and 2 < *R* < 5.

The severity of DILI was rate according to the livertox.nih.gov for DILI network severity score.^[7]^ Briefly, mild DILI (grade 1) was defined by elevated ALT and/or ALP but TBIL < 42.75 μmmol/L and INR < 1.5; Moderate DILI (grade 2) was defined by elevated ALT and/or ALP and TBIL ≥ 42.75 μmmol/L or INR ≥ 1.5; Moderate to severe DILI (grade 3) was defined by elevated ALT, ALP, TBIL and/or INR and hospitalization or ongoing hospitalization prolonged due to DILI; Severe DILI (grade 4) was defined by elevated ALT and/or ALP and TBIL ≥ 42.75 μmmol/L and at least one of the following criteria: (1) hepatic failure (INR > 1.5, ascites or encephalopathy), or (2) other organ failure due to DILI; Death or liver transplantation due to DILI was considered fatal DILI (grade 5).

The causality assessment followed the Roussel Uclaf Causality Assessment Method model.^[8]^

### 2.3. Statistical analysis

The statistical analyses were completed using the SPSS 29.0 software (IBM Corp., Armonk, NY). Categorical variables were expressed as frequencies and percentages. Continuous variables were presented as mean ± SD or median (range). Univariate analysis followed by multivariate logistic regression with forward stepwise selection (entry criterion: *P* < .05; removal criterion: *P* > .10) was conducted to identify risk factors associated with docetaxel-induced liver injury. Odds ratio (OR) and 95% confidence interval (CI) were calculated from the confidence and standard errors of the model, and Homos–Lemeshow test was used to evaluate the goodness of fit. Statistical significance was defined as a bilateral *P* value < .05. Receiver-operator characteristic curve analysis was performed to calculate the area under the receiver-operator characteristic curve (AUC). The best sensitivity and specificity were found through different cutoff points of the probability.

### 2.4. Ethical review and informed consent of patients

The study was approved by the Renmin Hospital Ethics Committee of Wuhan University (2018KC124). In view of the retrospective and observational nature of the study with no interventions performed, the need for informed consent was waived.

## 3. Results

### 3.1. Characteristics of patients with docetaxel-induced liver injury

In this study, a total of 2986 patients (746 males and 2240 females) admitted between January 2017 and April 2018 were identified through EMR review. Their average age were 54.36 years. A total of 1378 (46.15%, 1378/2986) patients with abnormal LFT met the criteria for liver dysfunction, according to CTCAE 5.0.^[9]^ Of those, 223 patients (40 males and 183 females) were diagnosed as docetaxel–DILI, with 27 patients of hepatocellular type, 82 patients of cholestatic type, and 114 patients of mixed type. The overall incidence of docetaxel–DILI was 7.47% (223/2986).

Of 223 patients, 102 patients (45.74%, 102/223) had DILI within 1 week, and 124 patients (55.61%, 124/223) had DILI within 4 weeks, 171 patients (76.68%, 171/223) had DILI within 8 weeks, and the mean time between initial exposure to docetaxel and subsequent docetaxel–DILI occurrence was 13.26 ± 16.31 days. Poor appetite (196/223, 87.89%), fatigue (154/223, 69.06%), jaundice (147/223, 65.92%), pruritus (96/223, 43.05%), nausea (62/223, 27.80%), abdominal distension (51/223, 22.87%), right upper type abdominal pain (22/223, 9.87%%), weigh loss (17/223, 7.62%), vomit (14/223, 6.28%), and fever (11/223, 4.93%), were the most frequent clinical presentations among docetaxel–DILI cases during the admission interval.

The distribution of DILI grades was 80 patients (35.87%, 80/223) with grade 1, 134 patients (60.09%, 134/223) with grade 2, 7 patients (3.14%, 7/223) with grade 3, 1 patient (0.45%, 1/223) with grade 4, and 1 patient (0.45%, 1/223) with grade 5.

With regards to outcome of DILI, 147 patients (65.92%, 147/223) were cured, 67 patients (30.04%, 67/223) were improved, 8 patients (3.59%, 8/223) were not improved, and 1 patient (0.45%, 1/223) were died.

### 3.2. Risk factors for docetaxel-induced liver injury during chemotherapy

Table 1 summarized differences in clinical and demographic variables between patients who did and did not develop DILI during receiving docetaxel therapy. Patients were divided into 2 groups based on the presence (n = 223) or absence (n = 2763) of DILI.

**Table 1:** Risk factors associated with the development of docetaxel-induced liver injury.

Variables	Non-DILI group	DILI group	Incidence of DILI (%)
Age			
<60	1885	146	7.19%
≥60	878	77	8.06%
Gender			
Female	2095	145	6.47%
Male	668	78	10.46%
Site of tumor			
Breast	1526	122	7.40%
Lung	468	34	6.77%
Ovary	198	18	8.33%
Head and neck	187	16	7.88%
Other sites	384	33	7.91%
BMI			
<18.5	690	59	7.88%
18.5–24	1101	83	7.01%
>24	962	91	8.64%
Drink			
No	2605	211	7.49%
Yes	158	12	7.06%
Smoke			
No	2512	210	7.71%
Yes	251	13	4.92%
NAFLD			
No	2634	219	7.68%
Yes	129	4	3.01%
HBV carrier			
No	2669	204	7.10%
Yes	94	19	16.81%
Diabetes			
No	2620	221	7.78%
Yes	143	2	1.38%
Chemotherapy			
DTX	1188	78	6.16%
DTX + CTX	462	4	0.86%
DTX + LBP	189	16	7.80%
DTX + DDP	313	27	7.94%
DTX + NDP	451	64	12.43%
DTX + CBP	52	8	13.33%
DTX + CAP	69	11	13.75%
DTX + EPI	39	15	27.78%

CAP = capecitabine, CBP = carboplatin, CI = confidence interval, CTX = cyclophosphamide, DDP = cisplatin, DILI = drug-induced liver injury, DTX = docetaxel, EPI = epirubicin, LBP = lobaplatin, NAFLD = no-alcoholic fatty liver disease, NDP = nedaplatin, OR = odds ratio.

As shown in Table 2, the univariate analysis showed these variables, such as gender (*P* < .001), HBV carrier (*P* < .001), diabetes (*P* = .012) and therapeutic regimen (*P* < .001), appeared to be related to DILI. However, on subsequent multivariate analysis, only HBV carrier (*P* = .004), diabetes (*P* = .007), and chemotherapy regimens (*P* < .001) were independently associated with DILI during receiving chemotherapy, respectively. Among them, diabetes (OR [95% CI] = 0.144 [0.035–0.591], *P* < .007) and docetaxel plus cyclophosphamide (OR [95% CI] = 0.132 [0.048–0.363], *P* < .001) were protective factors. However, HBV carrier (OR [95% CI] = 2.306 [1.315–4.045], *P* = .004), docetaxel plus nedaplatin regimen (OR [95% CI] = 2.024 [1.392–2.944], *P* < .001), docetaxel plus capecitabine regimen (OR [95% CI] = 2.296 [1.167–4.519], *P* < .016) and docetaxel plus epirubicin regimen (OR [95% CI] = 5.604 [2.946–10.658], *P* < .001), were the significant risk factors of docetaxel-induced liver injury, respectively.

**Table 2:** Logistic regression analysis of independent risk factors for docetaxel-induced liver injury.

Variable	Univariate analysis		Multivariate analysis	
	OR (95% CI)	*P* value	OR (95% CI)	*P* value
Age		.397		
<60	Ref.			
≥60	1.132 (0.849–1.509)			
Gender		<.001		.403
Female	Ref.		Ref.	
Male	1.687 (1.264–2.252)		1.154 (0.825–1.6123)	
Site of tumor		.943		
Breast	Ref.			
Lung	0.909 (0.613–1.347)	.634		
Ovary	1.137 (0.678–1.906)	.626		
Head and neck	1.070 (0.622–1.842)	.806		
Other sites	1.075 (0.720–1.604)	.724		
BMI		.356		
<18.5	0.882 (0.623–1.247)	.477		
18.5–24	Ref.			
>24	1.106 (0.786–1.557)	.563		
Drink		.834		
No	Ref.			
Yes	0.937 (0.513–1.714)			
Smoke		.103		
No	Ref.			
Yes	0.620 (0.349–1.101)			
NAFLD		.054		
No	Ref.			
Yes	0.373 (0.137–1.018)			
HBV carrier		<.001		.004
No	Ref.		Ref.	
Yes	2.645 (1.583–4.418)		2.306 (1.315–4.045)	
Diabetes		.012		.007
No	Ref.		Ref.	
Yes	0.166 (0.041–0.674)		0.144 (0.035–0.591)	
Chemotherapy		<.001		<.001
DTX	Ref.		Ref.	
DTX + CTX	0.132 (0.048–0.362)	<.001	0.132 (0.048–0.363)	<.001
DTX + LBP	1.289 (0.737–2.256)	.373	1.153 (0.645–2.062)	.632
DTX + DDP	1.314 (0.834–2.071)	.240	1 (0.599–1.669)	.999
DTX + NDP	2.161 (1.526–3.061)	<.001	2.024 (1.392–2.944)	<.001
DTX + CBP	2.343 (1.075–5.106)	.032	1.884 (0.836–4.249)	.127
DTX + CAP	2.428 (1.235–4.775)	.010	2.296 (1.167–4.519)	.016
DTX + EPI	5.858 (3.095–11.088)	<.001	5.604 (2.946–10.658)	<.001

CAP = capecitabine, CBP = carboplatin, CI = confidence interval, CTX = cyclophosphamide, DDP = cisplatin, DTX = docetaxel, EPI = epirubicin, LBP = lobaplatin, NAFLD = no-alcoholic fatty liver disease, NDP = nedaplatin, OR = odds ratio.

After that, we tested the combined effects of the 2 identified risk factors on the occurrence of docetaxel-induced liver injury. As shown in Table 3, with the increase of these risk factors, patients were associated with increased risk of docetaxel-induced liver injury occurrence in an exposure–response manner (*P* = .000), and cancer patients with 2 risk factors were associated with a 4.264-fold (95% CI = 1.580–11.505) increased risk.

**Table 3:** Combined effects of the defined risk factors of docetaxel-induced liver injury.

No. risk factors	Docetaxel-induced liver injury/patients	OR (95% CI)
0	119/2250	Ref.
1	99/710	2.902 (2.190–3.844)
2	5/26	4.264 (1.580–11.505)
*P*-value		.000

CI = confidence interval, OR = odds ratio.

Further analysis by the risk score and AUC showed that those factors contributed to an AUC of 0.693 (95% CI = 0.660–0.727), with a predictive sensitivity of 70.9% and specificity of 61.6%.

## 4. Discussion

This was a retrospective article to obtain data regarding the prevalence rates and causative drugs of DILI, which was executed in a single medical center by evaluating serum laboratory results related to liver toxicity. Because of the increased awareness of docetaxel-induced liver injury and the potential risk, resulting in fatal complications for cancer patients, a few groups have sought to determine the prevalence of clinical DILI. However, little is known about the incidence of docetaxel-induced liver injury in cancer patients. Up to now, only Wang et al, reported that they analyzed 647 metastatic breast cancer patients in admission, 67 of 647 (10.36%) of whom were confirmed to have docetaxel-induced liver injury.^[5]^ In the present study, we analyzed 2986 cancer patients in admission, 223 of 2986 (7.80%) of whom were confirmed to have docetaxel–DILI. This rate was relatively lower in the database of docetaxel-induced liver injury in cancer patients, compared with the early-reported incidence (10.36%).^[5]^ Similarly, it was also lower than the incidence of 15% for drug-induced liver injury caused by anticancer drugs reported in China.^[10]^

In the present study, the mean time between initial exposure to docetaxel and subsequent docetaxel-induced liver injury occurrence was 13.26 ± 16.31 days. This finding was similar to other study, which reported 14.01 ± 18.11 days.^[5]^ Poor appetite, fatigue, jaundice, and pruritus were the most frequent clinical presentations among docetaxel-induced liver injury cases during the admission interval.

A few reports^[11]^ have indicated that age was a potential risk factor for developing DILI, but this phenomenon was not found in this study, which may be related to the smaller sample size of those over 60 years old in this study. At present, there is not sufficient evidence to show that women have a higher risk of causing DILI by all drugs.^[2]^ However, in this study, women have a lower risk of liver injury caused by docetaxel. The available evidence does not support an increased susceptibility of diabetes and obesity to all-cause DILI.^[12]^ Interestingly, we reported that diabetes was a protected factor for docetaxel-induced liver injury (OR [95% CI] = 0.144 [0.035–0.591]).

HBV carrier proved to be an independent risk factor for docetaxel-induced liver injury in cancer patients. It was commonly assumed that underlying liver diseases are capable of increasing susceptibility to DILI.^[13–15]^ The presence of past HBV infection has been shown to cause an elevated risk of DILI as a result of drugs. In our study, there was a 2.31-fold increment for developing docetaxel-induced liver injury among past HBV infection cases (Table 2). Those data could support the hypothesis that HBV infection might interfere with intact immune function in liver injury.^[16]^ A previous study reported that nonalcoholic fatty liver disease could protect liver injury from the administration of statins.^[17]^ However, such a factor was not seen in docetaxel-induced liver injury. Therefore, the molecular mechanism of docetaxel-induced liver injury might differ with various drug exposure, where the drugs might initiate different pathogeneses.

Compared to the patients with single docetaxel exposure, patients with docetaxel plus epirubicin or docetaxel plus nedaplatin or docetaxel plus capecitabine combination chemotherapy were at increased risk of docetaxel-induced liver injury, while the opposite was true for the patients with docetaxel plus carboplatin combined chemotherapy (Table 2).

Clinically, timely discontinuation of the suspected drug causing liver injury and trying to avoid the reuse of the suspected or similar drugs as much as possible are the most main measures for the cause of liver injury and also the most basic treatment principle of DILI. The use of liver function protectants can help remove liver cells from harmed drugs and enhance the autoregeneration ability of cells. However, the combined application of 2 or more liver function protectants mainly to reduce ALT is not recommended. Only for patients with mixed DILI, it is acceptable to choose a drug mainly to reduce ALT and at the same time choose another drug to improve the manifestations of cholestasis.^[1]^ In the present study, docetaxel-induced liver injury was seen in 223 cases, and all cases were treated by liver protectants. In 137 of the 223 patients, liver function returned to normal values of AST and ALT within an average of 10.3 days. Seventy-nine patients showed slower declination of AST and ALT, at the average of 16.1 days. The remaining cases (7 patients) retained AST and/or ALT between grades I and II liver injury until 24 days, according to CTCAE 5.0. Non-recovery of liver function will cancel the consecutive chemotherapy.

As previously mentioned, the AUC for predicting liver injury risk was 0.693 in this study. The model has limited accuracy in identifying at-risk patients. To enhance utility, additional factors and improved statistical methods are needed.

However, there are some limitations in the study. First, the study may be limited by its single-center retrospective design. The single-center nature might restrict its generalizability. The available data is not enough or completely dependent on the case record, and researchers could not follow the patients and manage docetaxel-induced liver injury during hospitalization. Second, the study did not collect patients’ information on ethnic origin, genetic factors, income status, education status, treatment during, malnutrition and concomitant medications (e.g., hepatoprotectants), which might be risk factors for liver injury.

## 5. Conclusions

In the study, 223 (7.88%) patients were diagnosed as docetaxel-induced liver injury, among which the patients with ovarian cancer had the highest incidence rate (8.33%). HBV carrier and some therapeutic regimens might be potential risk factors for docetaxel-induced liver injury during hospitalization. Among these therapeutic combination regimens, docetaxel plus nedaplatin, docetaxel plus capecitabine and docetaxel plus epirubicin regimens could significantly increase the occurrence of docetaxel-induced liver injury during hospitalization, while docetaxel plus cyclophosphamide regime might be safer. HBV carriers might be more likely to have docetaxel-induced liver injury during receiving docetaxel plus nedaplatin, docetaxel plus capecitabine and docetaxel plus epirubicin regimens. Therefore, for patients with these risk factors, it might be advisable to actively consider optimum management to reduce the occurrence of docetaxel-induced liver injury, particularly hepatic function test.

## Acknowledgments

We thank to Department of Pharmacy, Renmin Hospital of Wuhan University for technical assistance.

## Author contributions

**Conceptualization:** Jian Yang.

**Data curation:** Haiyan Zhan, Yin Deng, Jing Xiang Yang, Jian Yang.

**Formal analysis:** Haiyan Zhan, Yin Deng, Caifei Huang, Jing Xiang Yang, Jian Yang.

**Investigation:** Haiyan Zhan, Yin Deng, Caifei Huang, Jing Xiang Yang, Jian Yang.

**Methodology:** Haiyan Zhan, Yin Deng, Caifei Huang, Jing Xiang Yang, Jian Yang.

**Project administration:** Jian Yang.

**Resources:** Jian Yang.

**Software:** Haiyan Zhan, Jian Yang.

**Supervision:** Jian Yang.

**Validation:** Jian Yang.

**Writing – original draft:** Haiyan Zhan, Yin Deng, Caifei Huang, Jing Xiang Yang, Jian Yang.

**Writing – review & editing:** Haiyan Zhan, Yin Deng, Caifei Huang, Jing Xiang Yang, Jian Yang.
